# Regioisomeric Effects on Enhancing *p*‐Type Characteristics of Self‐Assembled Molecules in Inverted Perovskite Solar Cells

**DOI:** 10.1002/advs.202521518

**Published:** 2026-01-29

**Authors:** Myeong‐Ho Hong, Sanwan Liu, Seong Chan Cho, Seung‐Joo Chang, Sang Uck Lee, Nam‐Gyu Park

**Affiliations:** ^1^ School of Chemical Engineering and Center for Antibonding Regulated Crystals Sungkyunkwan University Suwon Republic of Korea; ^2^ School of Chemical Engineering Sungkyunkwan University Suwon Republic of Korea; ^3^ SKKU Institute of Energy Science and Technology (SIEST) Sungkyunkwan University Suwon Republic of Korea

**Keywords:** dibromodibenzothiophene‐5,5‐dioxide, inverted, perovskite solar cell, regioisomeric effect, self‐assembled molecules

## Abstract

Although self‐assembled molecules (SAMs) have significantly advanced the development of inverted perovskite solar cells (PSCs), challenges remain in achieving uniform surface potential and reducing interfacial energy losses. Here, we introduced regioisomers with different bromine substitution sites, 3,7‐dibromodibenzothiophene‐5,5‐dioxide (3,7‐Br) and 2,8‐dibromodibenzothiophene‐5,5‐dioxide (2.8‐Br), as functional modulators for the widely used [4‐(3,6dimethyl‐9H‐carbazole‐9‐yl)butyl]phosphonic acid (Me‐4PACz). Compared with the meta‐substituted 3,7‐Br, the para‐substituted 2,8‐Br provides stronger *π*–*π* interactions with Me‐4PACz, enhanced *p*‐type characteristics, and a more favorable energy level alignment, thereby facilitating hole extraction. Moreover, the electron‐withdrawing yet electron‐rich sulfonyl moiety and bromo substituents in 2,8‐Br interact with perovskite, leading to more effective interfacial defect passivation and suppression of non‐radiative recombination. As a result, the power conversion efficiency (PCE) was enhanced from 23.40% to 25.40% after treatment of Me‐4PACz with the 2,8‐Br. In addition, the 2,8‐Br‐treated PSCs demonstrated improved light‐soaking stability under one sun illumination and thermal stability at 85°C, compared to the control device.

## Graphical abstract

Regioisomeric effects are a critical design parameter in self‐assembled monolyaer (SAM) engineering, underscoring their importance for optimizing buried interface properties and advancing the performance and stability of inverted perovskite solar cells.

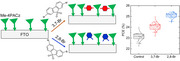

## 1 Introduction

Since the first report of solid‐state PSCs achieving a PCE of 9.7% with methylammonium lead iodide (MAPbI_3_) in 2012 [1], PSCs have attracted considerable attention owing to their low‐cost solution processability and outstanding optoelectronic properties. Over the past decade, substantial progress in materials composition, additive engineering, interface engineering, and device architecture has greatly enhanced the photovoltaic performance of PSCs [2, 3, 4, 5, 6]. As a result, a certified PCE exceeding 27% has been achieved [7]. Recently, the introduction of SAMs in inverted (p‐i‐n) PSCs has enabled higher performance compared to conventional (n‐i‐p) architectures. Most recent studies on inverted PSCs have adopted SAMs such as [4‐(3,6‐dimethyl‐9H‐carbazol‐9‐yl)butyl]phosphonic acid (Me‐4PACz), [2‐(3,6‐dimethoxy‐9H‐carbazol‐9‐yl)ethyl]phosphonic acid (MeO‐2PACz) and [2‐(9H‐carbazol‐9‐yl)ethyl]phosphonic Acid (2PACz) owing to their advantages, including efficient hole extraction, minimal parasitic absorption and low fabrication cost [8, 9].

However, these molecules tend to aggregate in solution due to their amphiphilic nature, resulting in non‐uniformity of the surface potential of SAMs [10]. Such a non‐uniform surface potential can induce interfacial recombination by allowing direct contact between the substrate and the perovskite layer [11]. In addition, the carbazole group oriented toward the perovskite layer has limited interaction with the underlying perovskite. These issues deteriorate the performance of inverted PSCs by reducing the open circuit voltage (*V*_OC_) and fill factor (FF), indicating the need for additional strategies to modulate SAMs [12, 13]. It was reported that *π*–*π* stacking could enhance interfacial quality [14, 15, 16]. However, the regioisomeric effects on *π*–*π* stacking, and consequently on the photovoltaic performance of inverted PSCs, have not yet been reported.

In this study, we present a SAM layer optimization strategy using regioisomers. The regioisomeric effects are investigated by introducing dibromodibenzothiophene‐5,5‐dioxide with different Br substitution sites through post‐treatment on the Me‐4PACz layer. The dibenzothiophene unit enables the incorporation of these molecules into the Me‐4PACz layer via *π*–*π* interactions, while their electron‐rich property is facilitated by the intramolecular electron‐withdrawing nature of ─SO_2_ and ─Br groups, which provide defect passivation of the bottom perovskite via coordination bonding. 2,8‐Br improves the uniformity of the surface potential and tunes the energy level of the Me‐4PACz layer, resulting in reduced interfacial recombination and enhanced charge extraction. Moreover, 2,8‐Br strongly interacts with the undercoordinated Pb^2+^ ion at the buried interface of the perovskite layer owing to its molecular geometry. The improved interfacial property leads to a substantial enhancement of device efficiency from 23.40% for the control device to 25.40% for the 2,8‐Br‐treated device. Moreover, the 2,8‐Br‐treated device demonstrates superior light‐soaking stability under 1 sun illumination and thermal stability at 85°C.

## 2 Results and Discussion

**FIGURE 1:**
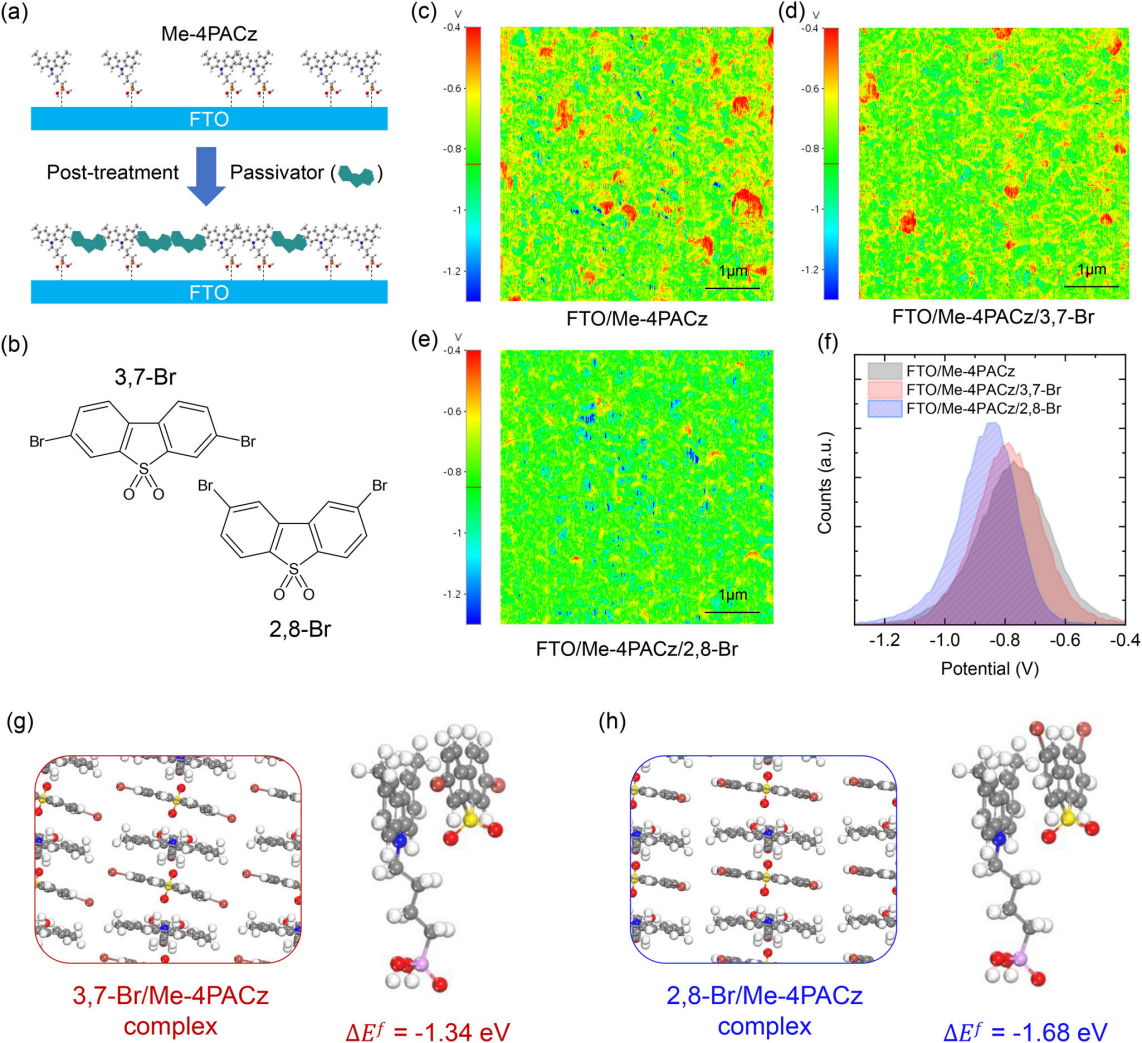
(a) Schematic illustration of the modification of the sparsely covered SAM layer by post‐treatment using regioisomers. (b) Chemical structure of 3,7‐Br and 2,8‐Br regioisomeric passivators. CPD mapping of (c) FTO/Me‐4PACz, (d) FTO/Me‐4PACz/3,7‐Br, and (e) FTO/Me‐4PACz/2,8‐Br films measured by KPFM and (f) their potential distribution. (g) Optimized structure and formation energy (Δ*E^f^*) of the 3,7‐Br/Me‐4PACz complex and (h) those of the 2,8‐Br/Me‐4PACz complex structure.

To modulate the non‐uniformity of the surface potential of SAMs, a passivator was post‐treated on the SAM layer. Figure 1a illustrates schematically an incompletely covered SAM layer on a fluorine‐doped tin oxide (FTO) conducting substrate and its subsequent passivation with a passivating agent by post‐treatment of the SAM layer. This modified Me‐4PACz layer is assumed to block direct contact between the substrate and the perovskite layer. Molecules bearing functional groups such as ─SO_2_, ─COOH, ─PO_x_, ─NH_2_, and ─OH can strongly interact with undercoordinated Pb^2+^ ions in the perovskite lattice through coordination of lone‐pair electrons, while groups such as ─Br and ─F can also interact, albeit more weakly, via halogen‐metal interactions or halogen bonding [17, 18, 19]. Based on this, we have selected regioisomers of 3,7‐Br and 2,8‐Br that contain both the SO_2_ and two Br atoms (Figure 1b). In addition, dibenzothiophene consists of three fused rings, similar to the carbazole unit in Me‐4PACz, which may facilitate its incorporation into the SAM layer through *π*–*π* interaction. To visualize electron cloud distribution, the electrostatic potential (ESP) of the molecules was calculated using density functional theory (DFT). As shown in Figure S1, both the ─SO_2_ group and the ─Br atoms exhibit negative ESP values. Among them, the ─SO_2_ group is expected to coordinate more strongly with undercoordinated Pb^2+^ at the perovskite bottom surface, owing to its higher electron density, thereby passivating iodine vacancy defects (V_I_).

We conducted Kelvin probe force microscopy (KPFM) measurements to examine how the post‐treated molecules on the Me‐4PACz layer affect the surface potential and energy level of the layer. As shown in the contact potential difference (CPD) mapping image of the FTO/Me‐4PACz film (Figure 1c), numerous red regions observed in the entire surface, corresponding to the FTO area with relatively high surface potential, are indicative of incomplete coverage of the Me‐4PACz. In contrast, the 3,7‐Br–treated film shows a noticeable but incomplete reduction in red regions (Figure 1d), whereas in the FTO/Me‐4PACz/2,8‐Br film, the red regions are substantially diminished and barely observable. The disappearance of the high‐potential domain suggests that the post‐treated molecules occupy the voids within the SAM film [20]. Since the mean CPD values are estimated to be −0.768, −0.782, and −0.863 V for the FTO/Me‐4PACz, FTO/Me‐4PACz/3,7‐Br, and FTO/Me‐4PACz/2,8‐Br films, respectively, the post‐treated molecules not only improve the uniformity of the surface potential but also increase the work function of Me‐4PACz. In addition, the *p*‐type property of Me‐4PACz is more strongly enhanced by 2,8‐Br than by 3,7‐Br, as evidenced by the greater increase in work function [21]. The change in work function further indicates the underlying chemical interaction between Me‐4PACz and the passivators. Hole transport and electron blocking are thus expected to be enhanced by the treatment with 3,7‐Br or 2,8‐Br [22]. The full width at half maximum (FWHM) of the CPD decreases after introduction of the passivators, indicating the formation of a more uniform interfacial layer with improved energy distribution (Figure 1f and Table S1). In addition, X‐ray photoelectron spectroscopy (XPS) analysis shows a characteristic peak of Br 3d in the Me‐4PACz/2,8‐Br sample, whereas no such peak appears in the pristine Me‐4PACz sample (Figure S2). This result confirms the successful deposition of 2,8‐Br on the Me‐4PACz surface.

To elucidate how 2,8‐Br improves the uniformity of the surface potential and coordination ability during perovskite growth relative to 3,7‐Br, we carried out density functional theory (DFT) simulations. We constructed SAM models of Me‐4PACz incorporating either 3,7‐Br or 2,8‐Br passivators (Figure 1g,h). The calculated formation energies (Δ*E_f_*) of 3,7‐Br/Me‐4PACz and 2,8‐Br/Me‐4PACz complexes reveal that 2,8‐Br exhibits a stronger interaction with Me‐4PACz (−1.68 eV) than 3,7‐Br (−1.34 eV), primarily through *π*–*π* interactions. This result is consistent with the enhanced surface potential observed experimentally upon 2,8‐Br treatment (Figure 1e). In addition, we examined the orientations of the passivators arising from the two possible directions of the sulfonyl (─SO_2_) group. As shown in Figure S3a, reversing the ─SO_2_ direction (toward perovskite) slightly changes the *π*–*π* distance but leaves the relative energies nearly unchanged. Consistently, DFT calculations for passivators on Me‐4PACz@FTO (110) show similar relative energies regardless of the ─SO_2_ orientation, indicating no strong preference for a single direction (Figure S3b). Furthermore, multiple non‐bonding interactions, including *O*⋅⋅⋅*H* hydrogen bonding and *Br*⋅⋅⋅*H* contacts, are observed in the optimized complexes (Figure S4), further stabilizing the passivator/Me‐4PACz assembly. These results suggest that, within the SAM layer, the passivators form stabilized complexes with Me‐4PACz (via *π*–*π* and non‐bonding interactions).

**FIGURE 2:**
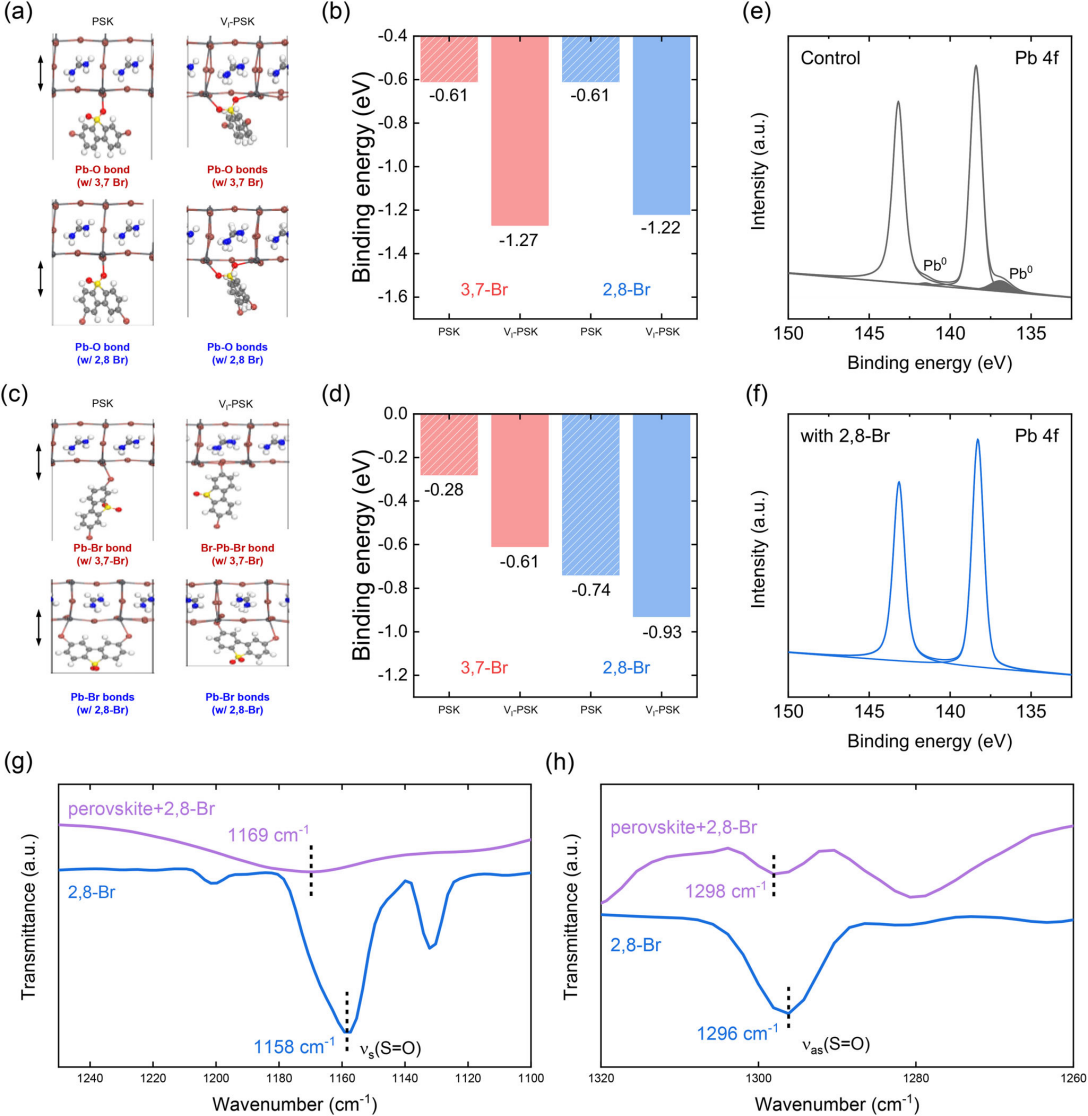
Optimized binding configurations and corresponding binding energies of passivators on pristine FAPbI_3_ perovskite (PSK) and I^−^ vacancy FAPbI_3_ (V_I_‐PSK) surfaces, showing (a,b) SO_2_‐PSK interactions and (c,d) Br–Pb coordination modes. XPS spectra of Pb 4f core level of the bottom surface of perovskites (e) without (control) and (f) with 2,8‐Br. (g,h) Partial enlarged FTIR spectra of the 2,8‐Br and the perovskite+2,8‐Br.

Furthermore, we investigated the role of passivators in defect passivation during the growth of bottom‐perovskite layers. As shown in Figure S5, we designed FAPbI_3_ surface structures with different terminations (FAI and PbI_2_) and calculated the I^−^ defect (V_I_) formation energy ($\Delta E_{\mathrm{def}}^{f}$). V_I_ formation was found to be more favorable on the PbI_2_‐terminated FAPbI_3_ surface than on the FAI‐terminated surface, with $\Delta E_{\mathrm{def}}^{f}$ = −0.46 eV. By calculating the binding energies (*∆*E_b_) of the passivators at the V_I_ site on the PbI_2_‐terminated FAPbI_3_ surface, we examined the interaction of the ─SO_2_ group with the bottom perovskite surface, as both passivators can orient the ─SO_2_ moiety toward the perovskite (Figure 2a). The optimized structures and binding energies indicate that sulfonyl anchoring commonly occurs in both passivators, contributing similarly to the interaction with the perovskite surface (Figure 2b). To further understand the origin of the different passivation ability depending on Br positions, we then considered the opposite orientation of passivators (Figure 2c). The calculated ∆E_b_ values in Figure 2d reveal that both passivators exhibit stronger binding at the defective surface than at the pristine one, indicating their potential for effective defect passivation. It is worth noting that 2,8‐Br shows higher binding affinity than 3,7‐Br, which can be attributed to the formation of multiple Pb─Br coordination bonds facilitated by its unique Br‐atom arrangement. These results indicate that sulfonyl‐based binding is a common feature, whereas the superior defect passivation of 2,8‐Br originates from its distinctive Br configuration, making it the more effective passivator candidate.

XPS and Fourier transform infrared spectroscopy (FTIR) measurements were conducted to further investigate the chemical interaction between 2,8‐Br and the perovskite. As shown in Figure 2e, the XPS spectra of the control sample display two main Pb 4f peaks at around 143.2 eV (Pb 4f_7/2_) and 138.4 eV (Pb 4f_5/2_), along with two small minor peaks at 141.5 and 136.9 eV corresponding to metallic lead (Pb^0^). These metallic unsaturated Pb^0^ indicate the presence of iodide vacancies and may be induced by undercoordinated Pb^2+^ species, which are known to act as recombination centers, deteriorating device performance [23, 24]. In contrast, the 2,8‐Br‐treated sample exhibits no detectable Pb^0^ peaks, suggesting a suppression of metallic lead formation (Figure 2f). This implies that the electron‐rich–SO_2_ groups in 2,8‐Br may coordinate with undercoordinated Pb^2+^ ions and passivate defects [25]. FTIR analysis provided complementary evidence for the interaction between 2,8‐Br and the perovskite. As shown in Figure 2g,h, symmetric (ν_s_) and asymmetric (ν_as_) stretching vibration of the sulfur‐oxygen double bond (S═O) in the pristine 2,8‐Br sample are observed at 1158 and 1296 cm^−1^, respectively [19]. Upon interfacing with the perovskite, these peaks shift to 1169 and 1298 cm^−1^, respectively, indicating a blueshift toward higher wavenumbers. This shift of the S═O stretching modes suggests a change in the local chemical environment, possibly due to coordination interaction with undercoordinated Pb^2+^ ions [26]. These XPS and FTIR results are in good agreement with the DFT calculations and experimentally demonstrate the chemical interaction between 2,8‐Br and the perovskite.

**FIGURE 3:**
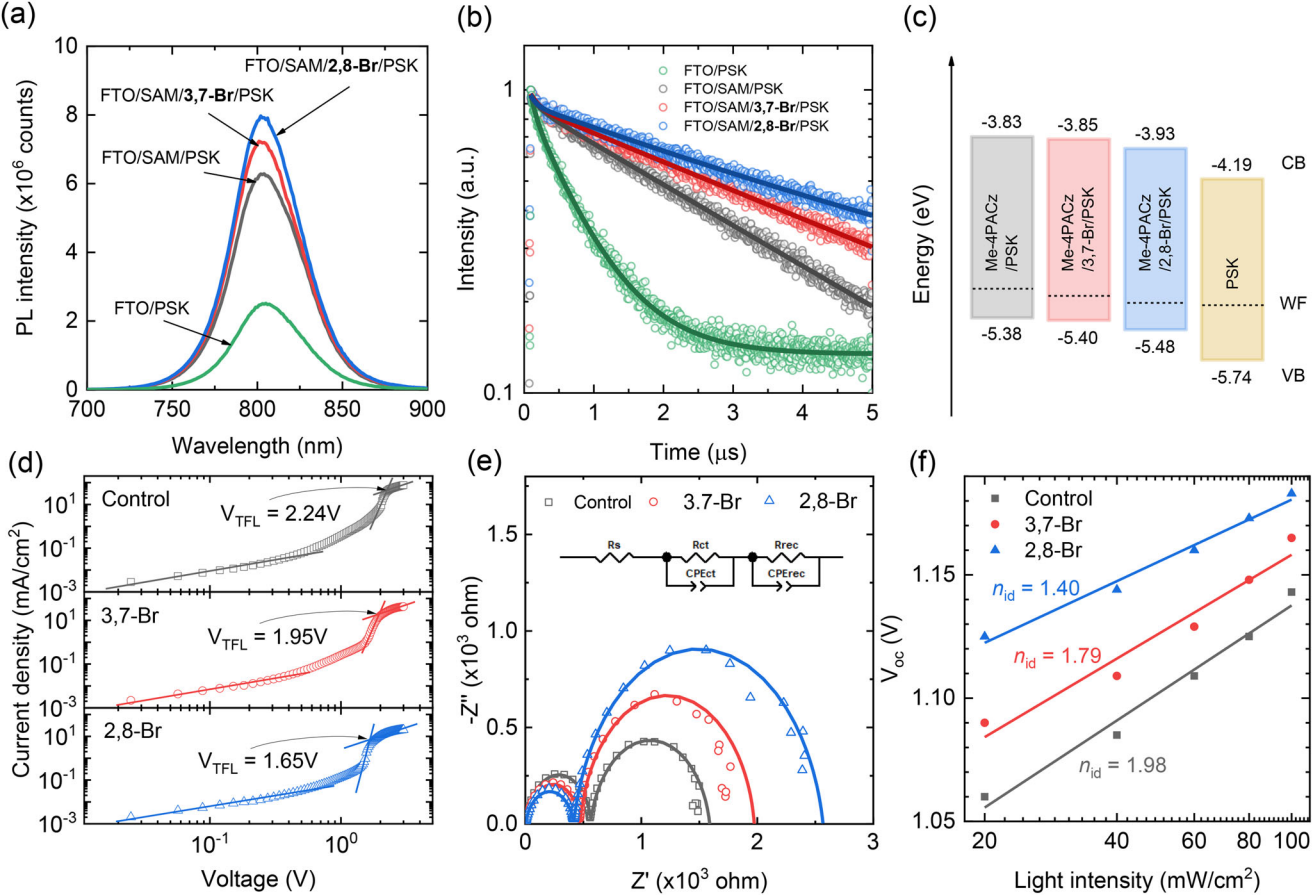
(a) SSPL and (b) TRPL spectra of the peeled‐off perovskite films deposited on FTO, FTO/SAM (= Me‐4PACz), FTO/SAM/3,7‐Br, and FTO/SAM/2,8‐Br. (c) Schematic energy levels diagram obtained from UPS and UV–vis analysis of the buried interface of the perovskite films based on different HTL conditions. (d) Dark *J*–*V* curves of the hole‐only devices employing the perovskite films deposited on the SAM layer without (control) and with post‐treatment with 3,7‐Br and 2,8‐Br. (e) Nyquist plot and (f) Light intensity dependent *V*_OC_ of the full devices employing the perovskite films deposited on the SAM layer without (control) and with post‐treatment with 3,7‐Br and 2,8‐Br. EIS was measured under one sun illumination with a bias voltage of 0.9 V (active area: 0.125cm^2^). n in (f) stands for ideality factor.

To further probe the influence of the passivators on the buried interfacial properties of perovskite, the perovskite layer was peeled off to expose its bottom surface (the peel‐off process is shown in Figure S6). Then we carried out steady‐state photoluminescence (SSPL) and time‐resolved photoluminescence (TRPL) measurements on peeled‐off perovskite layers deposited on different substrates (Figure 3a). The PL intensity of the control FTO/Me‐4PACz/perovskite film is higher than that of the FTO/perovskite film without Me‐4PACz, indicating that interfacial recombination is suppressed due to the passivation effect of Me‐4PACz [7, 22]. With additional molecular treatment using 3,7‐Br or 2,8‐Br, the PL intensity is further increased, which can be attributed to the improved uniformity of the surface potential and defect passivation at the bottom interface of the perovskite layer [20, 27, 28].

TRPL decay curves in Figure 3b were analyzed by fitting the data with the bi‐exponential decay equation of *I* (t) =  *I*_0_ + *A*_1_exp (− *t*/τ_1_) + *A*_2_exp (− *t*/τ_2_), where a fast decay component (τ_1_) and a slow decay component (τ_2_) are associated with charge extraction and non‐radiative recombination, respectively. The fitting results are listed in Table S2. Compared to the control film (τ_1_ = 141.3 ns, τ_2_ = 3177.1 ns), the films treated with 3,7‐Br and 2,8‐Br exhibit a decrease in τ_1_ (130.6 and 88.2 ns, respectively) and an increase in τ_2_ (4502.8 and 4684.0 ns, respectively), with 2,8‐Br proving more effective than 3,7‐Br. Average carrier lifetime (τ_avg_) is thereby extended from 2951 ns for the control to 4080 ns for 3,7‐Br and 4278 ns for 2,8‐Br, indicating that interfacial non‐radiative recombination is suppressed by the 2,8‐Br (3,7‐Br) treatment [20, 29], which is consistent with the transient photovoltage measurements (TPV) results (Figure S7).

Differential lifetime analysis was further conducted using the relation τ  =   − (dln [φ(*t*)]/*dt*)^−1^, where, φ(t) denotes the time‐dependent PL photon flux, which enables a clear distinction of carrier dynamics between charge transport and recombination feature (Figure S8) [30]. The rapid increase in the early time range (around 100 ns) reflects efficient charge transport, and the transition to plateau implies that Shockley–Read–Hall (SRH) recombination becomes dominant. Combining these data, it is suggested that this SAM modulation strategy not only facilitates charge extraction but also suppresses non‐radiative recombination through defect passivation.

Ultraviolet photoelectron spectroscopy (UPS) measurements were further performed on the bottom surface of the perovskite films based on different HTLs to explore the change in energy level arrangement induced by the passivators (Figure S9). Work function (W_F_) was calculated using the equation W_F_ = 21.22 eV—E_cutoff_, and the values increased for both 3,7‐Br‐treated and 2,8‐Br‐treated samples compared to the pristine Me‐4PACz/PSK sample, consistent with the KPFM results. Valence band maximum (VBM) was determined using the equation VBM = 21.22 eV—*E*_cutoff_ + *E*_onset_ [31, 32]. Then we obtained ultraviolet–visible (UV–vis) absorption spectra to investigate the effects of 3,7‐Br and 2,8‐Br treatment on absorptance and optical band gap of the perovskite layer (Figure S10). The optical band gap of the perovskite layer was estimated to be 1.55 eV from the Tauc plot. A schematic summary of the corresponding UPS data analysis is presented in Figure 3c. The VBM values of the buried interface of the perovskite films are shifted to deeper energy levels upon the passivator treatment, from −5.38 for Me‐4PACz/PSK to −5.40 for Me‐4PACz/3,7‐Br/PSK and to −5.48 for Me‐4PACz/2,8‐Br/PSK, thereby reducing energy offset between the buried interface and the perovskite. The increased W_F_ and reduced energy offset are expected to enhance the *p*‐type characteristics of the buried interface and facilitate efficient charge extraction from the perovskite layer [22].

To investigate the passivator‐induced defect‐passivation effects, defect‐mediated trap density (*n*_t_) was estimated from the space charge limited current (SCLC) measurement using a hole‐only device structure (FTO/Me‐4PACz (without and with 3,7‐Br or 2,8‐Br)/perovskite/spiro‐MeOTAD/Ag), combined with capacitance–frequency (C–F) data from electrochemical impedance spectroscopy (EIS) measurement using FTO/Me‐4PACz (without and with 3,7‐Br or 2,8‐Br)/perovskite/Au structure. Trap‐filled limit voltage (*V*_TFL_) was determined from the intersection point of slopes of the trap‐filled limited region (n ≥ 2) and the Child region (n = 2). As shown in Figure 3d, the *V*_TFL_ values decrease from 2.24 V (control) to 1.95 V (3,7‐Br) and 1.65 V (2,8‐Br), which implies that *n*_t_ decreases since *n*_t_ is proportional to *V*_TFL_. Based on the formula *V_TFL_* =  q*n_t_L* ^2^/2ε_0_ε [33, 34], where *q*, *L*, *ε*_0_, and *ε* are the electric charge (1.602 × 10^−19^ C), thickness of the perovskite layer (780 nm), the vacuum permittivity (8.8542 × 10^−14^ F/cm), and dielectric constant of the perovskite, respectively, we estimated *n*_t_. The dielectric constant *ε* for each sample was determined using geometric capacitance (*C*_g_) at 1.0608 × 10^4^ Hz derived from C‐F plots (Figure S11) [35]. The relevant parameters for calculating *n*_t_ are listed in Table S3. The estimated *n*_t_ decreases from 5.74 × 10^15^/cm^3^ for the control to 4.76 × 10^15^/cm^3^ for 3,7‐Br and 3.60 × 10^15^/cm^3^ for 2,8‐Br, confirming the passivation effect and demonstrating the superior effectiveness of 2,8‐Br.

EIS was further analyzed under one sun illumination, which was combined with light intensity‐dependent *V*_OC_. The Nyquist plot obtained from EIS in Figure 3e shows two semicircles, where the first semicircle in the high frequency region and the second semicircle in the low frequency region are associated with charge transfer resistance (*R*_ct_) and recombination resistance (R_rec_), respectively [36]. The fitted parameters using an equivalent circuit are listed in Table S4. Compared to the control device, both 3,7‐Br‐ and 2,8‐Br‐treated devices exhibit reduced series resistance (R_s_) and R_ct_, together with increased R_rec_, indicating enhanced charge extraction and suppressed interfacial recombination [37]. Notably, the 2,8‐Br‐treated device shows a more evident enhancement. These EIS results are well matched with the KPFM and PL analyses. These improvements in the buried interface properties are attributed to enhanced uniformity of the surface potential, better energy‐level alignment, and effective defect passivation, all of which are expected to increase *V*_OC_ and FF. As shown in Figure 3f, the ideality factor of the diode (*n*_id_) derived from light intensity‐dependent *V*_OC_ decreases from 1.98 for the control to 1.79 for 3,7‐Br and 1.40 for 2,8‐Br, indicating a reduction of trap‐assisted interfacial recombination [38, 39].

**FIGURE 4:**
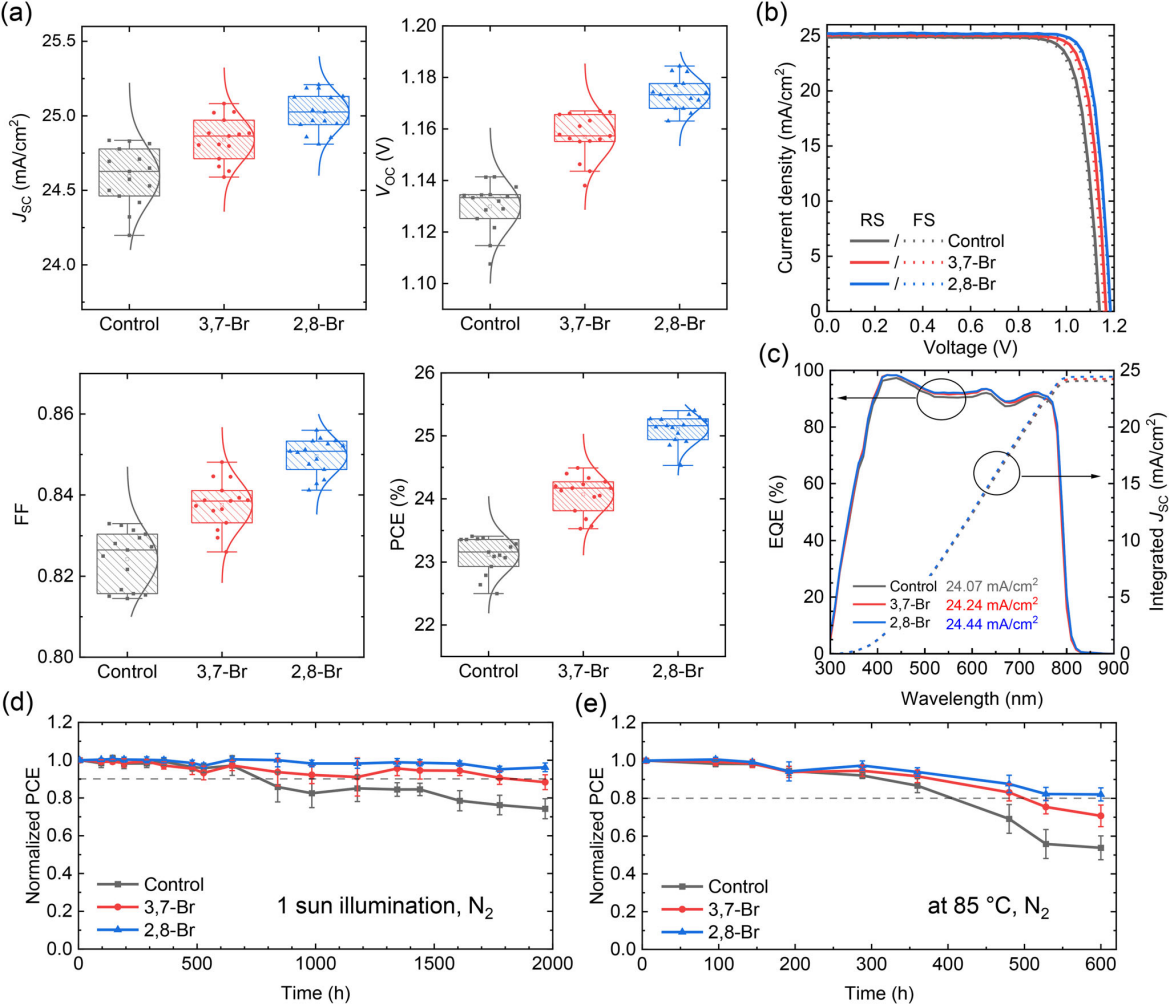
(a) Statistical photovoltaic parameters of the control devices, 3,7‐Br‐treated devices, and 2,8‐Br‐treated devices. (b) Reverse‐ and forward‐scanned *J*–*V* curves of the champion control devices, 3,7‐Br‐treated devices, and 2,8‐Br‐treated devices (c) EQE data and integrated *J*_SC_ of the champion devices. (d) Light soaking stability and (e) thermal stability of the control devices, and 3,7‐Br devices and 2,8‐Br devices.

The impact of the SAM modulation strategy on the photovoltaic performance of PSCs was evaluated. Figure 4a shows the statistical photovoltaic parameters for devices with different passivators. The control devices exhibit a mean PCE of 23.11 ± 0.28% with corresponding mean *J*_SC_, *V*_OC_, and FF of 24.60 ± 0.19 mA/cm^2^, 1.13 ± 0.01 V, and 82.42 ± 0.68%, respectively. Compared to the control devices, 3,7‐Br‐treated devices show an improved mean PCE of 24.07 ± 0.29% with *J*_SC_, *V*_OC_, and FF of 24.84 ± 0.14 mA/cm^2^, 1.16 ± 0.01 V, and 83.76 ± 0.57%. Further enhancement is observed from the 2,8‐Br‐treated devices, demonstrating a mean PCE, *J*_SC_, *V*_OC_, and FF of 25.11 ± 0.22%, 25.02 ± 0.13 mA/cm^2^, 1.17 ± 0.01 V, and 84.96 ± 0.43%, respectively. It is worth noting that *V*_OC_ and FF are obviously improved by the passivator treatment. The current density‐voltage (*J*–*V*) curves of champion devices under reverse and forward scanning are illustrated in Figure 4b. The champion performance extracted from the statistical data shows that the PCE increases from 23.40% (control) to 24.54% with 3,7‐Br post‐treatment, and further to 25.40% with 2,8‐Br. These enhancements are mainly attributed to an increase in *V*_OC_ from 1.139 V (control) to 1.166 V (3,7‐Br) and 1.185 V (2,8‐Br) and FF from 82.61% (control) to 84.02% (3,7‐Br) and 85.08% (2,8‐Br). The photovoltaic parameters of the champion devices are listed in Table S5. As shown in Figure 4c, integrated *J*_SC_ values estimated from external quantum efficiency (EQE) spectra were 24.07 mA/cm^2^ for the control, 24.24 mA/cm^2^ for the 3,7‐Br‐treated, and 24.44 mA/cm^2^ for the 2,8‐Br‐treated device, respectively. Compared to *J*_SC_ values obtained from the *J*–*V* curve, the ratios were 96.8% for the control, 96.8% for the 3,7‐Br‐treated, and 97.0% for the 2,8‐Br‐treated device, which fall within the accepted deviation range of 5% [40, 41]. In addition, to validate the universality of our strategy, we applied it to devices based on other SAM (2PACz) and perovskite composition (FA_0.95_Cs_0.05_PbI_3_). The best‐performing 2,8‐Br‐treated devices exhibited high *V*_OC_ and FF similarly, suggesting the good applicability of this approach (Figure S12). Notably, the 2,8‐Br‐treated devices exhibit improved light soaking stability, retaining 96.2% of their initial PCEs after 1968 h in a N_2_‐filled glovebox, compared to 88.3% for the 3,7‐Br‐treated devices and 74.3% for the control devices (Figure 4d). A similar trend is observed in the thermal stability test, where the control devices degrade significantly, maintaining only 53.8% of their initial PCE, whereas the 3,7‐Br‐ and 2,8‐Br‐treated devices retained 70.7% and 82.1% of their initial PCE, respectively (Figure 4e). Thermogravimetric analysis (TGA) supports the enhanced thermal stability of the 2,8‐Br‐treated devices. The temperature of 2,8‐Br@5% weight loss is 280.1°C, which is much higher than the practical application of solar cells (Figure S13). Device stabilities under 1 sun illumination and thermal stress (85°C) were finally investigated. As shown in Figure S14, the 2,8‐Br‐treated devices retained 84.1% of initial PCE after 480 h in a N_2_‐filled glovebox, while the 3,7‐Br‐treated and control devices maintained 73.7% and 60.1%, respectively. The improved stability of the 2,8‐Br‐treated devices under both light and thermal stress further highlights the critical role of buried interface modification. These enhancements are attributed to uniformity of the surface potential, effective suppression of interfacial recombination, and reduction of undercoordinated Pb^2^^+^ defects. Based on the regioisomeric effects observed in this work, it should be emphasized that both the chemical interaction between Me‐4PACz and the organic passivator, as well as the passivator–defect interaction within the perovskite, must be carefully considered when selecting passivators for the perovskite/HTL interface in inverted PSCs.

## 3 Conclusions

In this work, we investigated the impact of SAM modulation through molecular treatment on the Me‐4PACz layer. Two regioisomers of dibromobenzothiophene derivatives containing a sulfonyl group were employed as candidate materials for incorporation into the Me‐4PACz layer via *π*–*π* interaction. Experimental and theoretical results confirmed that treatment with 3,7‐Br and 2,8‐Br enhanced the *p*‐type characteristics of the HTL and effectively passivated defects at the bottom perovskite interface. Importantly, the regioisomeric effect played a decisive role: 2,8‐Br not only achieved superior uniformity of the surface potential and facilitated hole extraction, but also strongly suppressed interfacial recombination through coordination with undercoordinated Pb^2+^ ions, owing to its favorable molecular geometry. Consequently, the PCE of 23.40% was significantly improved to 25.40% after surface treatment of Me‐4PACz with 2,8‐Br. In addition, the molecular treatments enhanced both light‐soaking and thermal stability. Overall, this study demonstrates that regioisomeric effects are a critical design parameter in SAM engineering, underscoring their importance for optimizing buried interface properties and advancing the performance and stability of inverted perovskite solar cells.

## Conflicts of Interest

The authors declare no conflicts of interest.

## Supporting information




**Supporting File**: advs74105‐sup‐0001‐SuppMat.docx

## Data Availability

The data that support the findings of this study are available from the corresponding author upon reasonable request.
