# Supplementary material for: Regioisomeric Effects on Enhancing p‐Type Characteristics of Self‐Assembled Molecules in Inverted Perovskite Solar Cells
Source: Adv Sci (Weinh). 2026 Jan 29;13(19):e21518. doi: 10.1002/advs.202521518 (PMC13045227; doi:10.1002/advs.202521518)
Supplement: Supplementary file 1 — Supporting File: advs74105‐sup‐0001‐SuppMat.docx [file ADVS-13-e21518-s001.docx]

*Supporting information for*

**Regioisomeric Effects on Enhancing p-Type Characteristics of Self-Assembled Molecules in Inverted Perovskite Solar Cells**

Myeong-Ho Hong^1^, Sanwan Liu^1*^, Seong Chan Cho^2^, Seung-Joo Chang^1^, Sang Uck Lee^2*^, Nam-Gyu Park^1,3*^

^1^School of Chemical Engineering and Center for Antibonding Regulated Crystals, Sungkyunkwan University, Suwon 16419, Republic of Korea

^2^School of Chemical Engineering, Sungkyunkwan University, Suwon 16419, Republic of Korea

^3^SKKU Institute of Energy Science and Technology (SIEST), Sungkyunkwan University, Suwon 16419, Republic of Korea

*Corresponding authors: liusanwan@skku.edu (S.L.); suleechem@skku.edu (S.U.L.); npark@skku.edu (N.-G.P.)

**Material synthesis**

Formamidine iodide was synthesized by reacting 15 g of formamidine acetate (99%, Sigma-Aldrich) with 22.8 ml of hydriodic acid (HI) (57% w/w aqueous solution) in an ice bath for 2 hours. The remaining solvent was removed using a rotary evaporator at 80 °C for 2 h. The brown powder was dissolved in ethanol (99.5%, Samchun) and recrystallized by pouring in diethyl ether (DEE) (99.0%, Samchun). The purification process was repeated until a white powder was obtained. Methylammonium iodide (MAI) was synthesized by reacting 15 ml of HI with 55 ml of methylamine (ca. 9% in isopropyl alcohol, ca. 2 mol/l, TCI) in an ice bath for 1 h. The residual solvent was removed using a rotary evaporator at 60 °C for 1 h. The same recrystallization and purification procedure as used for FAI was applied. Formamidinium lead triiodide (FAPbI_3_) was synthesized by reacting 3.30g of FAI with 8.85g of lead iodide (PbI_2_, Acros) in 12ml of 2-Methoxyethanol (99.8%, Sigma-Aldrich). The mixture was heated at 125 °C for 1 h, resulting in precipitation of FAPbI_3_ powder. The product was washed 5 times with acetonitrile (99.9%, Daejung) and DEE. All the synthesized powders were dried under vacuum for over 12 h and stored in an Ar-filled glove box.

**Device Fabrication**

Fluorine-doped tin oxide (FTO) glass substrates were sequentially cleaned by sonication in detergent, deionized water, acetone, and isopropyl alcohol (IPA) for 15 min each. The cleaned substrates were treated with UV-ozone for 1 hour and transferred to a N_2_-filled glovebox. To deposit SAMs, 0.5 mg of Me-4PACz (>99.0%, TCI) was dissolved in 1 ml of ethanol, and 180 μl of the solution was dropped on the substrate. After 15 seconds, the substrate was spin-coated at 3000 rpm for 30 s (acceleration rate: 600 rpm/s), followed by annealing at 100 °C for 10 min. Residual unreacted Me-4PACz was removed by rinsing with ethanol, and the substrate was annealed again at 100 °C for 10 min. For additional molecular treatment, 3,7-dibromodibenzothiophene-5,5-dioxide (3,7-Br, >97.0%, TCI) solution and 2,8-dibromodibenzothiophene-5,5-dioxide (2,8-Br, >95.0%, TCI) solution were prepared by dissolving 0.5 mg of each compound in 1 ml of toluene, respectively. 180 μl of the 3,7-Br solution was statically dropped on the substrate and then spin-coated at 3000 rpm for 30 s (acceleration rate: 600 rpm/s) after 5 s. The substrate was then annealed at 100 °C for 10 min. The 2,8-Br was deposited using the same method as 3,7-Br. To prepare perovskite precursor solution, 0.9147 g of synthesized FAPbI_3_, 0.0221 g of cesium iodide (99.999%, Sigma-Aldrich), 0.0270 g of MAI, 0.1959 g of PbI_2_ (99.99%, TCI) and 0.0155 g of methylammonium chloride (>99.99%, Greatcellsolar) were dissolved in 800 μl of dimethyl formamide (99.8%, Sigma-Aldrich) and 200 μl of dimethyl sulfoxide (99.9%, Sigma-Aldrich). 60 μl of the precursor solution was dropped on the substrate and spread uniformly. The film was spin-coated in a two-step process: 1000 rpm for 10 s (acceleration rate: 200 rpm/s), followed by 6000 rpm for 30 s (acceleration rate: 1000 rpm/s). 180 μl of anisole (99.7%, Sigma-Aldrich) was dropped 10 s before the end of the second step and followed by annealing at 120 °C for 20 min. After cooling down to room temperature, the top surface of the perovskite layer was passivated using piperazine dihydroiodide (PDI, >98.0%, TCI) and phenethylammonium iodide (PEAI, Great cell solar) solution. PDI and PEAI solutions were prepared by dissolving 3 mg of PDI in 3.6 ml of IPA and 4 mg of PEAI in 2 ml of IPA, respectively. 80 μl of each solution was sequentially spin-coated at 4000 rpm for 25 s dynamically. Residual solvent was evaporated and removed by annealing at 120 °C for 5 min. All the chemical solution was filtered by a 0.20 μm pore-sized PTFE filter (Hyundati MICRO) before use. Finally, 25 nm of fullerene, 6 nm of bathocuproine, and 100 nm of Ag were deposited sequentially via thermal evaporator.

**Characterizations**

Current density–voltage (*J-V*) curve measurement was carried out under AM 1.5G one sun illumination (100 mW/cm^2^) using a solar simulator (Oriel Sol 3A, class AAA) equipped with a 450 W Xenon lamp (Newport 6280NS) and a Keithley 2400 source meter. The light intensity was calibrated using a NREL-certified Si reference cell with a KG-5 filter. The illuminated area was limited to 0.125 cm^2^ with a metal mask during measurement. Photoluminescence (PL) and time-resolved photoluminescence (TRPL) spectra were measured using an FLS1000 (Edinburgh Instruments). TRPL spectra were measured using time-correlated single-photon counting (TCSPC) with a 405 nm beam source. Absorbance and transmittance spectra measurements were conducted using a UV-visible spectrometer (Lambda 45, PerkinElmer). Optical band of the perovskite films. To obtain the space-charge-limited current (SCLC), dark J-V were measured using FTO/Me-4PACz/without and with additional molecular treatment/perovskite/spiro-MeOTAD/Ag devices. Electrochemical impedance spectra (EIS) and capacitance-frequency (C-F) measurements were carried out using an Autolab 302B. The devices were covered with a metal mask (aperture area: 0.125 cm^2^) and illuminated under one sun at a bias voltage of 0.9 V during measurement. The dielectric constant was extracted from the plateau region observed at 1.0608×10^4^ Hz using the formula $\varepsilon_{r}=\frac{C\times d}{\varepsilon_{0}\times A}$. The external quantum efficiency (EQE) measurement was conducted using a QEX-7 series EQE system (PV measurement Inc.) under AC mode, where equipped with a 75 W xenon lamp chopped by a monochromator. Kelvin probe force microscopy (KPFM) measurement was conducted using an atomic force microscope (AFM, NX10, Park system) with an NSC14 cantilever. Ultraviolet photoelectron spectroscopy (UPS) spectra and X-ray photoelectron spectroscopy (XPS) spectra were obtained from NEXSA G2 (Thermo Fisher Scientific) with a He I (21.22 eV) source for UPS and an Al K_α_ X-ray source for XPS. Scanning electron microscopy (SEM) images of the bottom surface of the perovskite layer were obtained from a JSM-7600F (JEOL) instrument. Thermogravimetric analysis (TGA) was conducted using the STA200R model (Hitachi). Fourier transform infrared (FTIR) spectra were obtained from an IRTracer-100 (Shimadzu) spectrometer using attenuated total reflectance (ATR) mode.

**Computational detail**

All density functional theory (DFT) calculations were performed using the Gaussian 09 program package and the Vienna *ab initio* simulation package (VASP 5.4.4) for molecular and periodic systems.^1-5^ For molecular systems, geometry optimizations and evaluations of key molecular properties, including dipole moments, atomic charges, and electrostatic potentials, were performed on 2,8-Br compared to 3,7-Br using Gaussian 09 at the B3LYP/6-311G level of theory. Optimized geometries were confirmed as energy minima by the absence of imaginary vibrational frequencies. For periodic systems, VASP calculations employed the projector augmented wave (PAW) method^4-7^ with exchange-correlation interactions treated using the Perdew–Burke–Ernzerhof (PBE) functional within the generalized gradient approximation (GGA).^8^ Brillouin zone integrations were conducted using Monkhorst-Pack k-point grids of 2$\times$2$\times$1 for passivator/Me-4PACz slab and FAPbI_3_ surface structures, respectively.^9^ Dispersion interactions were accounted for using the DFT-D3 correction method.^8,10^ Geometry optimizations were conducted using a plane-wave cutoff energy of 500 eV, with convergence criteria of residual atomic forces below 0.04 eV/Å. Detailed information about the structural design process and calculations is provided in Supplementary Note S1.

**Note S1. DFT simulation detail for FAPbI_3_ surface structure**

To examine the surface properties of FAPbI_3_ for 3,7-Br and 2,8-Br binding, we designed surface models of FAPbI_3_ with a 15 Å vacuum gap in the z-direction, which prevented interlayer interactions. Two surface terminations, divided by FAI and PbI_2_, respectively, were considered to investigate the surface properties. The bottom two layers of FAPbI_3_ surfaces were fixed to represent their bulk properties. Their thermodynamic stability is determined by calculating surface energy ($\gamma_{\mathrm{FAPb}I_{3}}$), which is defined by,

|  | $\gamma_{\mathrm{FAPb}I_{3}}=(E_{\mathrm{FAPb}I_{3}}^{FAI/PbI_{2}\mathrm{termination}}-E_{\mathrm{FAPb}I_{3}}^{\mathrm{Bulk}}*n)/ (2*A)$ | Eqn. S1 |
| --- | --- | --- |

, where $E_{\mathrm{FAPb}I_{3}}^{FAI/PbI_{2}\mathrm{termination}}$ is the total energy of the fully relaxed surface structure with FAI or PbI_2_ termination, n is the number of bulk units to fit the stoichiometry of the surface slab model (in this study, n = 12), $E_{\mathrm{FAPb}I_{3}}^{\mathrm{Bulk}}$ is the total energy of the FAPbI_3_ bulk structure, and A is the surface area.

In addition, to estimate the possibility of I^-^-defect frequently observed in the halide-based perovskite structure, we eliminated the I atom from the perovskite (FAPbI_3_) surface and calculated I^-^-defect formation energy ($\Delta E_{\mathrm{def}}^{f}$) with the following equation.

|  | ${\Delta E}_{\mathrm{def}}^{f}=(E_{I^{-} defect FAPbI_{3}}^{\left( + \right)}-E_{\mathrm{FAPb}I_{3}}+E_{I}^{(-)})$ | Eqn. S2 |
| --- | --- | --- |

, where $E_{I^{-} defect FAPbI_{3}}^{\left( + \right)}$ is the total energy of the positively charge perovskite surface with I^-^ defect, $E_{\mathrm{FAPb}I_{3}}$ is the total energy of defect-free perovskite surface, $E_{I}^{(-)}$ indicates the total energy of the negatively charged single I^-^ ion (the charge state is determined by charge the “NELECT” option in VASP code). Subsequently, we also investigated the binding of the 3,7-Br and 2,8-Br molecules to the iodide defect site of the FAPbI_3_ surface using molecular binding energy (${\Delta E}_{b}$). This was calculated using the following equation:

|  | ${\Delta E}_{b}=(E_{Molecule@I^{-}defect FAPbI_{3}}^{FAI/\mathrm{PbI}_{2}\mathrm{termination}}-E_{I^{-}defect FAPbI_{3}}^{FAI/\mathrm{PbI}_{2}\mathrm{termination}}-E_{\mathrm{Molecule}})$ | Eqn. S3 |
| --- | --- | --- |

, where $E_{Molecule@I^{-}defect FAPbI_{3}}^{FAI/\mathrm{PbI}_{2}\mathrm{termination}}$ is the total energy of the fully relaxed FAPbI_3_ surface with molecules on the surface, and $E_{I^{-}defect FAPbI_{3}}^{FAI/\mathrm{PbI}_{2}\mathrm{termination}}$ is the total energy I^-^-defect FAPbI_3_ surface, $E_{\mathrm{Molecule}}$ is also the total energy of the 3,7-Br and 2,8-Br molecules.

**Figure S1.** Molecular structure and electrostatic potential (ESP) distribution of (a) 3,7-Br and (b) 2,8-Br.

**Figure S2.** XPS spectra of Br 3d core level of the Me-4PACz and Me-4PACz/2,8-Br films.

**Figure S3.** (a) Relative energies of optimized passivator–Me-4PACz complexes considering molecular orientation and π–π interaction distance. (b) Relative energy of passivator incorporated Me-4PACz@FTO (110) surface structures.


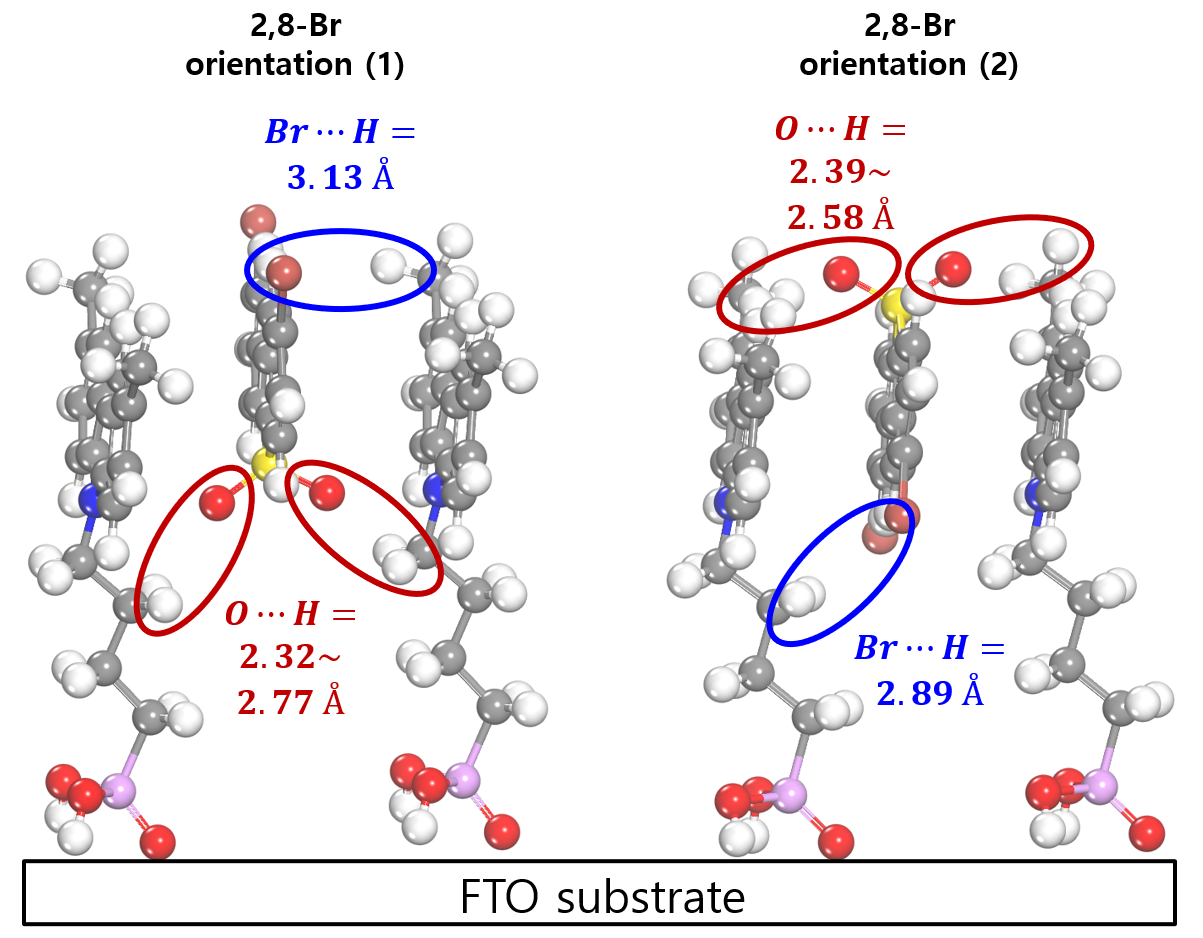


**Figure S4.** Non-bonding interactions via hydrogen bond and their distances in the optimized 2,8-Br/Me-4PACz complex.

**Figure S5.** Calculated I^-^ defect formation energy ($\Delta E_{\mathrm{def}}^{f}$) for FAPbI_3_ (100) surfaces with PbI_2_ termination (upper panel) and FAI termination (lower panel), where $\gamma$ denotes the corresponding surface energy.

**Figure S6**. The illustration of the peel-off method for exposing the buried interface of the perovskite film. A small amount of UV-curable glue is pasted on the top surface of the prepared perovskite film. Then, the substrate is covered with the glass side of another substrate. After 8 hours, the substrate is cured by UV light. And then, the bottom surface of the perovskite film is peeled off mechanically from the substrate.

**Figure S7.** TPV decay curves of the devices employing perovskite films deposited on the SAM layer without (control) and with post-treatment with 3,7-Br and 2,8-Br. The device structure was FTO/Me-4PACz /Perovskite/PDI/PEAI/C60/BCP/Ag.

**Figure S8.** Differential lifetime derived from the fits to the transients in TRPL for the perovskite (PSK) films deposited on the SAM (= Me-4PACz) without and with post-treatment with 3,7-Br and 2,8-Br.

**Figure S9**. UPS spectra for (a) Me-4PACz/PSK, (b) Me-4PACz/3,7-Br/PSK, (c) Me-4PACz/2,8-Br/PSK, and (d) PSK films.

**Figure S10.** (a) UV-vis absorption spectra and (b) Tauc plot of the perovskite films deposited on FTO, FTO/SAM (= Me-4PACz), FTO/SAM/3,7-Br, and FTO/SAM/2,8-Br.

**Figure S11.** Capacitance-frequency (C-f) plot of the FTO/Me-4PACz (without and with 3,7-Br or 2,8-Br)/perovskite/Au devices under one sun at bias of 0.9 V, measured over the frequency range from 0.1 Hz to 1MHz.

**Figure S12**. The *J-V* curves of the champion devices based on (a) 2PACz/FA_0.85_MA_0.1_Cs_0.05_PbI_3_ and (b) Me-4PACz/FA_0.95_Cs_0.05_PbI_3_.

**Figure S13**. Thermogravimetric analysis (TGA) curves of the 2,8-Br.

**Figure S14**. The stability of the control, 3,7-Br-treated, and 2,8-Br-treated devices under both 1 sun illumination and 85 °C thermal stress in a N_2_-filled glovebox.

**Table S1.** CPD mean values and FWHM of FTO/Me-4PACz, FTO/Me-4PACz/3,7-Br and FTO/Me-4PACz/2,8-Br films

| Sample | mean CPD (V) | FWHM |
| --- | --- | --- |
| Me-4PACz | -0.768 | 0.2713 |
| 3,7-Br | -0.782 | 0.2434 |
| 2,8-Br | -0.863 | 0.2111 |

**Table S2.** The fitted time constant from TRPL results of the perovskite layer on different substrate. Carrier lifetime (τ_avg_) is derived from the equation $\tau_{avg}=(A_{1}\tau_{1}+A_{2}\tau_{2})/(A_{1}+A_{2})$.

| Sample | *A*_1_ | *τ*_1_ (ns) | *A*_2_ | *τ*_2_ (ns) | *τ*_avg_ (ns) |
| --- | --- | --- | --- | --- | --- |
| w/o HTL | 0.113 | 84.5 | 0.712 | 692.0 | 608.8 |
| Control | 0.071 | 141.3 | 0.876 | 3177.1 | 2950.9 |
| 3,7-Br | 0.093 | 130.6 | 0.866 | 4502.8 | 4080.2 |
| 2,8-Br | 0.075 | 88.2 | 0.774 | 4684.0 | 4278.4 |

**Table S3**. Parameters of geometric capacitance (C_g_), thickness of the perovskite layer (L), electric charge (ε), trap-filled limit voltage (V_TFL_), and trap density (n_t_) for control, 3,7-Br treated, and 2,8-Br treated samples.

| Sample | *C*_g_ (×10^-8^ F) | *L* (×10^-7^cm) | *ε* | *V*_TFL_ | *n*_t_ (×10^15^ cm^-3^) |
| --- | --- | --- | --- | --- | --- |
| Control | 2.0 | 780 | 14.1 | 2.24 | 5.74 |
| 3,7-Br | 1.9 | 780 | 13.4 | 1.95 | 4.76 |
| 2,8-Br | 1.7 | 780 | 12.0 | 1.65 | 3.60 |

**Table S4**. The fitted parameters from EIS measurement for the control, 3,7-Br treated, and 2,8-Br treated device (aperture area: 0.125cm^2^).

| Sample | R_s_ (Ω) | R_ct_ (Ω) | CPE_ct_ (F) | R_rec_ (Ω) | CPE_rec_ (F) |
| --- | --- | --- | --- | --- | --- |
| Control | 11.25 | 552.7 | 5.92×10^-8^ | 1026 | 8.88×10^-5^ |
| 3,7-Br | 8.388 | 464.6 | 6.10×10^-8^ | 1501 | 7.57×10^-5^ |
| 2,8-Br | 1.946 | 416 | 1.02×10^-7^ | 2149 | 8.49×10^-5^ |

**Table S5.** Photovoltaic parameters of champion device for the control, 3,7-Br treated, and 2,8-Br treated samples.

| Sample | Scan direction | J_SC_ (mA/cm^2^) | V_OC_ (V) | FF (%) | PCE (%) |
| --- | --- | --- | --- | --- | --- |
| Control | reverse | 24.87 | 1.139 | 82.61 | 23.40 |
|  | forward | 24.86 | 1.133 | 81.90 | 23.07 |
| 3,7-Br | reverse | 25.05 | 1.166 | 84.02 | 24.54 |
|  | Forward | 25.01 | 1.159 | 83.30 | 24.15 |
| 2,8-Br | reverse | 25.19 | 1.185 | 85.08 | 25.40 |
|  | forward | 25.16 | 1.182 | 84.85 | 25.23 |

References

1. M. J. Frisch, G. W. Trucks, H. B. Schlegel, G. E. Scuseria, M. A. Robb, J. R. Cheeseman, G. Scalmani, V. Barone, B. Mennucci, G. A. Petersson, H. Nakatsuji, M. Caricato, X. Li, H. P. Hratchian, A. F. Izmaylov, J. Bloino, G. Zheng, J. L. Sonnenberg, M. Hada, M. Ehara, K. Toyota, R. Fukuda, J. Hasegawa, M. Ishida, T. Nakajima, Y. Honda, O. Kitao, H. Nakai, T. Vreven, J. A. Montgomery Jr., J. E. Peralta, F. Ogliaro, M. Bearpark, J. J. Heyd, E. Brothers, K. N. Kudin, V. N. Staroverov, R. Kobayashi, J. Normand, K. Raghavachari, A. Rendell, J. C. Burant, S. S. Iyengar, J. Tomasi, M. Cossi, N. Rega, J. M. Millam, M. Klene, J. E. Knox, J. B. Cross, V. Bakken, C. Adamo, J. Jaramillo, R. Gomperts, R. E. Stratmann, O. Yazyev, A. J. Austin, R. Cammi, C. Pomelli, J. W. Ochterski, R. L. Martin, K. Morokuma, V. G. Zakrzewski, G. A. Voth, P. Salvador, J. J. Dannenberg, S. Dapprich, A. D. Daniels, Ö. Farkas, J. B. Foresman, J. V. Ortiz, J. Cioslowski, D. J. Fox, *Gaussian 09, Revision E.01*; Gaussian, Inc., Wallingford, CT 2009.

2. G. Kresse, J. Hafner, Ab initio molecular dynamics for open-shell transition metals, *Phys. Rev. B* **1993**, *48*, 13115.

3. G. Kresse, J. Hafner, Ab initio molecular-dynamics simulation of the liquid-metal–amorphous-semiconductor transition in germanium, *Phys. Rev. B* **1994**, *49*, 14251.

4. G. Kresse, J. Furthmüller, Efficiency of ab-initio total energy calculations for metals and semiconductors using a plane-wave basis set, *Comput. Mater. Sci.* **1996**, *6*, 15.

5. G. Kresse, J. Furthmüller, Efficient iterative schemes for ab initio total-energy calculations using a plane-wave basis set, *Phys. Rev. B* **1996**, *54*, 11169.

6. P. E. Blöchl, Projector augmented-wave method, *Phys. Rev. B* **1994**, *50*, 17953.

7. G. Kresse, D. Joubert, From ultrasoft pseudopotentials to the projector augmented-wave method, *Phys. Rev. B* **1999**, *59*, 1758.

8. J. P. Perdew, K. Burke, M. Ernzerhof, Generalized gradient approximation made simple, *Phys. Rev. Lett.* **1996**, *77*, 3865.

9. H. J. Monkhorst, J. D. Pack, Special points for Brillouin-zone integrations, *Phys. Rev. B* **1976**, *13*, 5188.

10. S. Grimme, J. Antony, S. Ehrlich, H. Krieg, A consistent and accurate ab initio parametrization of density functional dispersion correction (DFT-D) for the 94 elements H–Pu, *J. Chem. Phys.* **2010**, *132*, 154104.
